# Effect of Grit-blasting on the Color Stability of Zirconia Ceramics Following Exposure to Beverages

**DOI:** 10.7759/cureus.7170

**Published:** 2020-03-03

**Authors:** Samer Alaqeel

**Affiliations:** 1 Dental Health, King Saud University, Riyadh, SAU

**Keywords:** zirconia, color stability, grit-blasting, beverages, cielab, nbs

## Abstract

Aim

The aim of this study was to evaluate the effect of grit-blasting on the color stability of zirconia ceramics following exposure to beverages.

Method

A total of 80 planar zirconia specimens were prepared from dense sintered zirconium blocks and were randomly allocated into four groups (n=20). Ten specimens from each group were grit-blasted (GB) using aluminum trioxide powder from a distance of 10 mm. The remaining 10 specimens were used as such without grit-blasting (NGB). The grit-blasted and non-grit-blasted specimens were immersed in artificial saliva, Coffee, Red Bull, and Coca-Cola at room temperature over a 28-day test period. Color measurement (ΔE) of the zirconia specimens was performed at baseline (T0) and after seven (T1), 14 (T2), and 28 (T3) days of immersion in the beverages using the Color Eye 7000A Spectrophotometer (Gretag Macbeth, New Windsor, NY). The data were analyzed using analysis of variance (ANOVA) and the post-hoc Bonferroni test. A p-value of less than 0.05 was considered statistically significant.

Result

The GB specimens immersed in coffee showed the highest color changes as compared to other groups (4.47± 0.04) and the NGB specimens stored in saliva showed the lowest ΔE values (0.83± 0.03). Energy drinks and soft drinks showed similar ΔE values at the end of the 28-day study period in both the NGB and GB groups. All the specimens, irrespective of the beverages, in both groups showed increased ΔE values at different immersion periods except for NGB specimens immersed in saliva, which showed decreased ΔE values from T2 to T3.

Conclusion

GB specimens showed increased ΔEs compared to NGB Zirconia ceramics. Among the beverages, the coffee immersion of GB specimens showed marked color changes. The specimens immersed in the energy and acidic drinks from both groups showed perceivable color changes at the end of the 28-day study period.

## Introduction

There is always a constant quest for the use of aesthetic biomaterials in restorative dentistry. In the last decade, zirconium dioxide (ZrO_2_) or zirconia (commonly, yttria-stabilized) are the widely used aesthetic material in restorative dentistry. That said, the increased use of zirconia is due to their phenomenal properties, such as superior strength, relatively low elastic modulus, moderate fracture toughness, wear resistance, and biocompatibility [1-2]. Yttria (3 mol%) is added to biomedical grade zirconia to stabilize the crystal structure transformation during sintering thereby improving their mechanical properties [3].

The inert characteristics of zirconia ceramic are the acid-resistant polycrystalline structure, which results in weak bonding to resin composites [4]. Hence, it is recommended to roughen the zirconia surface to create an irregular surface to allow the free movement of resin composites into the irregularities. This aspect is highly applicable for orthodontic bonding in which the orthodontic brackets are retained onto the roughened zirconia surface by resin composites. Grit blasting with alumina particles is the most effective method for the surface roughening of zirconia-based ceramics with the least damage to the surface [5].

On the contrary, rough surface on a dental restorative surface can predispose to the accumulation of plaque, residues, and stains resulting in diminished gloss and discoloration of the restoration [6]. Although the discoloration of the dental restorative surface does not have a role in the physiological success, it could influence the overall acceptance by the patient. Thus, special attention is required considering the role played by the dietary contents in the color stability of dental restorative materials, especially with the consumption of carbonated beverages, energy drinks, alcohol, tea, and coffee [7].

The consumption of energy drinks has remarkably increased over the last few years with more than 10% of students consuming energy drinks regularly. The ingredients in most of the commercially available energy drinks are based on simple sugars, caffeine, taurine, inositol, B vitamins, glucuronolactone, and plant extracts. The positive effects of consuming energy drinks have mixed scientific study outcomes where some studies prove the positive effect and others do not [8].

Similar to energy drinks, the consumption of soft drinks has also increased tremendously among all the age groups in both developed and developing countries. The term "soft drinks" includes all types of drinks except alcoholic ones, either carbonated or non-carbonated. These soft drinks contain various types of acids such as tartaric acid, lactic acid, maleic acid, and phosphoric acid [9]. The detrimental effect of soft drinks is determined by the acids present in soft drinks, i.e., phosphoric acid-based drinks cause more damage than citric acid-based beverages [10]. The entry point of these beverages into the human body is through the oral cavity and the effect these beverages have on the oral cavity should not be overlooked.

Color is one such phenomenon that plays an important role in aesthetic dentistry. The color changes in dental restorative materials upon exposure to simulated oral environments has been the topic of extensive research. The perception of color has mesmerized human beings from ancient days. Color is the result of the interaction of three main determinants: a) light source, b) physical properties of the object, and c) the observer. Any discrepancy in any of the three factors can change the perception of the color [11].

Consequently, the present study aimed to evaluate the effect of grit blasting on the color stability of zirconia ceramics following exposure to common beverages.

## Materials and methods

The materials and beverages used in the current study are presented in Table 1.

**Table 1:** Materials and beverages used in the study

Name	Composition	Manufacturer
Everest^® ^ZH-Blank	Dense sintered, yttrium-stabilized HIP (hot isostatic pressing) zirconium blocks	KaVo, Germany
Korox™	Aluminum trioxide powder, purity: > 95%	Bego, Bremen, Germany
Nescafé^®^ GOLD (Staining solution)	Skimmed milk powder, glucose syrup, instant coffee, coconut oil, lactose, acidity regulator, stabilizer, salt, anti-caking agent, natural flavoring	Nestle Middle East Manufacturing LLC, Dubai
Red Bull^®^ (Energy drink)	Sucrose, glucose, acidity regulatory sodium, caffeine, vitamins, natural flavors, colors	Red Bull GmbH, Austria
Coca-Cola® (Soft drink)	Carbonated water, sugar, caffeine, phosphoric acid, caramel color, natural flavoring	The Coca-Cola Company, Saudi Arabia

Specimen preparation

A total of 80 specimens (6.0 mm × 6.0 mm x 5 mm) identical in dimensions were obtained from commercially available dense sintered zirconium blocks. The blocks were sectioned with a diamond saw (IsoMet™, Buehler, IL) under running water. The obtained specimens were then polished using 1000-grit silicon carbide abrasive paper and cleaned under running tap water. The cleaned specimens were randomly allocated into four groups according to the immersion procedures. Ten specimens from each group were grit-blasted (GB) and the remaining 10 specimens were used as such (NGB). The specimens were grit-blasted using alumina powder (50 µm, 2.5 bar, 10 s) from a distance of approximately 10 mm by a hand device (LEMAT NT4, Wassermann, Hamburg, Germany) held perpendicular to the zirconia surface. Following grit-blasting, the specimens were cleaned in an ultrasonic unit for approximately 10 minutes.

The surface roughness of the grit blasted and non-grit-blasted groups were determined using a 3-D optical non-contact surface profilometer (Bruker Contour GT, Tucson, AZ). This device utilizes a nano lens Atomic Force Microscopy (AFM) module with a fully automated turret with programmable X, Y, and Z movements providing high-resolution surface data. For the same purpose, five scans at 1 mm distance were performed on three randomly selected specimens from each group. Scanning electron microscope (SEM) photomicrographs of the grit-blasted and non-grit-blasted specimens were performed to observe the surface topographic changes following grit-blasting. The SEM (JEOL JSM-5900 LV SEM, Tokyo, Japan) was operated at 20 kV, at a high vacuum, and at 200x magnification.

Immersion procedure

The zirconia specimens were immersed in the respective beverages (Artificial Saliva, Coffee, Red Bull, and Coca-Cola) for a period of 28 days. The coffee solution was prepared by adding 15 g of coffee powder to 250 ml of hot water and stirring well until it cooled down to oral temperature (37°C). The coffee was filtered to remove any residue and stored in an airtight amber-colored bottle [12]. The coffee was freshly prepared daily before immersion, and the fresh can of Red Bull and Coca-Cola was used daily for immersion. Adequate quantity (25 ml) of the beverages was maintained in all groups during the immersion procedure. Following each immersion period, the zirconia specimens were thoroughly cleaned with a soft brush under running water and stored in artificial saliva for the remainder of the time. The artificial saliva was prepared based on the description from a previous study [13]. The pH of the prepared saliva was estimated to be 7.5 and was periodically changed daily during the time of the study.

Color measurement

Color measurements were performed at baseline (T0) and after seven (T1), 14 (T2), and 28 (T3) days of immersion. All the specimens were removed from the solutions, cleaned under tap water and dried using tissue paper prior to measurement. The color measurement was performed using benchtop Color Eye 7000A spectrophotometer (Gretag Macbeth, New Windsor, NY, USA) calibrated against white background according to manufacturer’s recommendations. The color changes were determined using Commission Internationale de l’Eclairage L*a*b* (CIELab) color space system. The CIE L*a*b* color system is a chromatic value color space that measures the value and Chroma on L*a*b* coordinates. The total color differences (∆E*) were calculated using the formula:

\(∆E= [(∆L*)^2^+(∆a*)^2^+(∆b*)^2^]^1/2^\)

L* measures the lightness of the color from black to white, a* measures the color in the red and green dimensions, and b* measures color in the yellow and blue dimensions. For each specimen, five measurements at a 1 mm distance were performed and the average corresponded to the CIElab values of that specimen. In relating the color values to the clinical environment, the ∆E values were converted to the National Bureau of Standards (NBS) units using the below formula [14].

$NBS unit = 0.92 &times; &Delta;𝐸$

The clinical interpretation of the obtained NBS units are presented in Table 2.

**Table 2:** National Bureau Standards (NBS) interpretation of color changes

NBS unit	Color change	Clinical interpretation
0.0–0.5	Trace	Extremely slight change
0.5–1.5	Slight	Slight change
1.5–3.0	Noticeable	Perceivable
3.0–6.0	Appreciable	Marked change
6.0–12.0	Much	Extremely marked change
>12.0	Very much	Change to another color

Statistical analysis

All the data analysis was performed using Statistical Package for the Social Sciences v.18 (IBM Corporation, Armonk, NY) statistical software. The data obtained were analyzed by applying Kruskal-Wallis and posthoc Bonferroni tests. A p-value of less than 0.05 was considered statistically significant.

## Results

The effect of grit-blasting on the surface roughness (Ra) of zirconia specimens is shown in Table 3.

**Table 3:** Mean surface roughness (Ra) of the groups Different superscript alphabet within a column indicates a significant difference (*p* < 0.05).

Groups	Surface roughness (in µm)
Non-grit-blasted	0.27 ± 0.0068^a^
Grit-blasted	0.78 ±0.0081^b^

**Figure 1:**
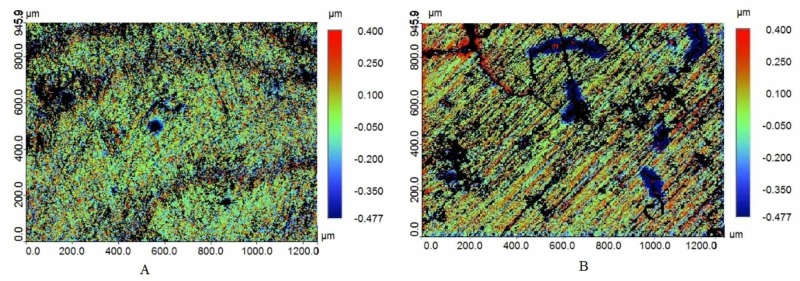
Profilometery images of the specimens A) Non-grit-blasted and B) Grit-blasted

The mean surface roughness of the non-grit-blasted and grit-blasted specimens was 0.27 μm and 0.78 μm respectively. A significant difference in the mean surface roughness was observed between the groups (*p *< 0.05). Profilometry surface roughness images of the non-grit-blasted and grit-blasted specimens zirconia specimens are presented in Figure 1.

**Figure 2:**
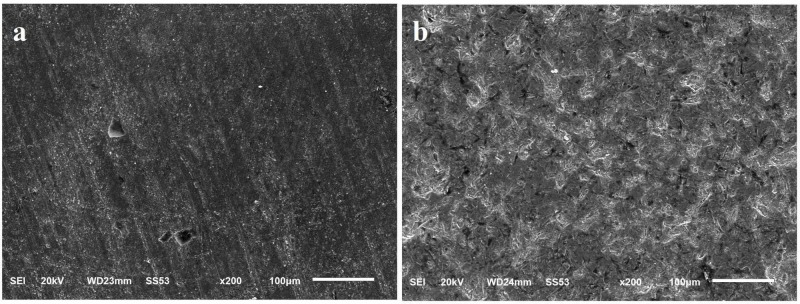
Scanning electron microscopy (SEM) photomicrographs (200x) of the zirconia specimen a) Non-grit-blasted and b) Grit-blasted

The SEM images of the non-grit-blasted and grit-blasted specimen’s zirconia specimens are shown in Figure 2. The microphotographs demonstrated significant surface changes of the zirconia specimens following grit-blasting.

**Figure 3:**
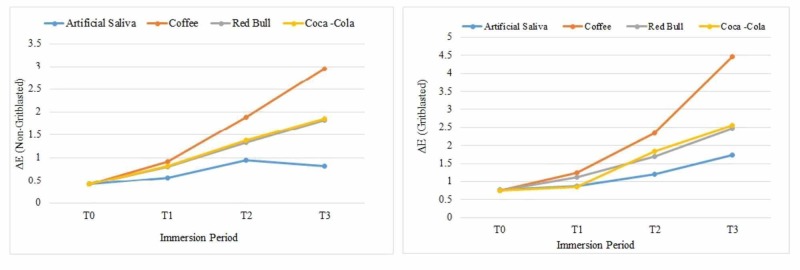
Mean ∆E values of the non-grit-blasted and grit-blasted groups

The mean ΔE values of the non-grit-blasted and grit-blasted groups are presented in Figure 3.

Significant differences in the ΔE values were observed for the zirconia specimens following grit-blasting (p<0.05). Also, different immersion periods contributed to significant differences in the ΔE values (p<0.05). The GB specimens immersed in coffee showed the highest color changes compared to other groups (4.47 ± 0.04) and the NGB specimens stored in saliva showed the lowest ΔE values (0.83 ± 0.03). Energy drinks and soft drinks showed similar ΔE values at the end of the 28-day study period in both NGB and GB groups. All the specimens irrespective of the beverages in both groups showed increased ΔE values at different immersion periods except for NGB specimens immersed in saliva. These specimens showed decreased ΔE values from T2 to T3 (Table 4).

**Table 4:** ∆E of the groups at different immersion periods. The values are expressed as Mean (SD). Different superscript alphabets within a column indicate a significant difference between the beverages. Different superscript numerals within a row indicate a significant difference between the GB and NGB for that particular beverage.

Immersion period	Artificial saliva		Coffee		Red Bull		Coca-Cola	
	NGB	GB	NGB	GB	NGB	GB	NGB	GB
T0	0.42^a,1^±0.01	0.77^a,2^±0.01	0.42^ a,1^ ±0.01	0.76^ a,2^±0.01	0.42^ a,1^ ±0.01	0.76 ^a,2^±0.02	0.42^ a,1^±0.016	0.76^ a,1^±0.01
T1	0.57^b,1^±0.01	0.87^b,2^±0.02	0.92^ b,1^±0.02	1.24^ b,2^±0.01	0.81^ b,1^±0.03	1.13^ b,2^±0.02	0.83^ b,1^±0.049	0.85^ b,1^±0.03
T2	0.95^c,1^±0.02	1.21^c,2^±0.02	1.89^ c,1^±0.04	2.35^ c,2^±0.02	1.33^ c,1^±0.03	1.70 ^c,2^±0.03	1.38^ c,1^±0.037	1.85^ c,3^±0.02
T3	0.83^d,1^±0.03	1.73^d,2^±0.02	2.96^ d,1^±0.09	4.47^ d,2^±0.04	1.83^ d,1^ ±0.02	2.47 ^d,2^±0.05	1.85^ d,1^±0.037	2.55^ d,4^±0.01

The NBS interpretation of the calculated ΔE values is presented in Table 5.

**Table 5:** National Bureau of Standards (NBS) interpretation of calculated ΔE values

Beverages	Groups	ΔE values	NBS units	Clinical interpretation
Artificial saliva	NGB	0.83	0.76	Slight change
	GB	1.73	1.59	Perceivable
Coffee	NGB	2.96	2.72	Perceivable
	GB	4.47	4.11	Marked change
Red Bull	NGB	1.83	1.68	Perceivable
	GB	2.47	2.27	Perceivable
Coca-Cola	NGB	1.85	1.70	Perceivable
	GB	2.55	2.34	Perceivable

The grit-blasted specimen immersed in the coffee solution showed clinically marked color changes compared to other groups. On the contrary, NGB specimens stored in saliva showed a slight change after 28 days of storage. The rest of the specimens showed clinically perceivable color changes.

## Discussion

The current study evaluated the effect of grit-blasting on the color stability of zirconia ceramics following exposure to common beverages. Although previous studies have evaluated the color stability of zirconia ceramics, this is the first attempt to study the effect of grit blasting on the color stability of zirconia [15-18]. This was important because the acid-resistant polycrystalline structure of zirconia is resistant to etching and results in weak bonding to resin composites [4]. Therefore, it becomes necessary to roughen the zirconia surface before bonding to resin composite especially in the orthodontic bonding of adult patients. Surface roughening promotes micro-mechanical retention between the resin composites and the zirconia surface.

Previous studies have shown that grit-blasting is an efficient surface roughening process, with the least damage to the zirconia surface [19]. Grit-blasting increases the surface roughness and the wettability of the material thereby enhancing the bond strength between the resin composite and the zirconia specimen [20]. However, the increased surface roughness in grit-blasted zirconia surfaces has some drawbacks such as reduced mechanical integrity and promoting biofilm adhesion [4].

It has been reported that ceramic materials especially zirconia are hydrophobic and exhibit better color stability than composite resins. Hence, there is no much literature comparing the color stability of ceramics and composite resins [8,21]. The outcome of the study demonstrated that the grit-blasted zirconia surface did significantly affect the color values of the zirconia surface irrespective of the beverages used for immersion. The GB specimens showed increased ∆E units compared to NGB specimens at all immersion period. The specimens were immersed for 5 min, three times daily, thus representing a medium frequency of beverage intake by an individual [22]. The highest ∆E (4.47 ±0.04) values were obtained for GB specimens stored in the coffee solution.

The coffee solution was used as a staining solution as it is the most chromogenic substance compared to other beverages used in the study [23]. The constituents of coffee such as tannin and chlorogenic acid are demonstrated to cause discoloration. The pH of coffee ranging from 4.9-5.2 can further worsen the discoloration process. Coffee is among the world's most widely consumed beverages in spite of being one of the causes for discoloration of the tooth and restorative materials [24]. Following the 28-day study period, GB specimens stored in the coffee solution showed marked changes compared to other specimens. Similarly, for zirconia specimens immersed in the coffee solution in previous studies, higher than clinically acceptable values were reported [15-16].

The consumption of energy and acidic soft beverages has remarkably increased over the last decades. Previous studies have confirmed the positive role of these beverages in staining tooth and restorative materials [8]. In the current study, a significant difference in the ∆E values of the GB and NGB specimens was observed when immersed in the energy and soft drinks (p<0.05). However, the energy and soft drinks had little impact on the color stability of the zirconia specimens whether grit-blasted or not. This could be explained by the acid-resistant property of zirconia ceramics, which could have shown less vulnerability for discoloration. Similar to the outcome of the present study, Colombo et al. also observed clinically acceptable color values with zirconia specimens following one week of storage in a Coca-Cola beverage [15]. Furthermore, in a study by Derafshi et al., it was found that zirconia specimens stored in mouth rinses exhibited clinically acceptable ∆E values [17].

The GB and NGB specimens immersed in the artificial saliva showed the least ∆E values among the other beverages. The ∆E values increased from baseline to 28-day study period in both the GB and NGB groups. However, the ∆E values decreased from T2 to T3 in NGB specimens.

The ∆E values obtained in the present study were converted to NBS units for relating the laboratory outcome to the clinical environment. The ΔE values between 1 and 3 are perceptible to the naked eye and ΔE values above 3.3 are critical and clinically unacceptable [25]. The ΔE values of GB and NGB specimens immersed in different beverages were interpreted to have perceivable color changes (NBS units 1.5-3.0) but GB specimens immersed in coffee showed marked changes (NBS units 4.11). The only specimens to have shown slight color changes were NGB specimens stored in artificial saliva.

The major limitation of the current study was in-vitro design. This said the restorative materials could behave differently in the oral cavity and, moreover, the toothbrushing and salivary actions are found to modify the color stability of the restorative materials. The color measurements in this study were obtained under a white background, however, the values could vary under different backgrounds.

## Conclusions

The GB specimens showed an increased ΔEs compared to NGB zirconia ceramics. Among the beverages, the coffee immersion of GB specimens showed marked or clinically unacceptable color changes. The specimens immersed in the energy and acidic drinks from both groups showed perceivable color changes at the end of the 28-day study period. Future studies should be directed toward confirming the outcome of the present study with the clinical environment. Furthermore, the effect of grit-blasting distances on the color stability of the zirconia surfaces needs to be investigated.

## References

[1] Lee JH, Lee M, Kim KN, Hwang CJ (2015). Resin bonding of metal brackets to glazed zirconia with a porcelain primer. Korean J Orthod.

[2] Lung CYK, Matinlinna JP (2010). Resin bonding to silicatized zirconia with two isocyanatosilanes and a cross-linking silane. Part I: experimental. Silicon.

[3] Bona AD, Pecho OE, Alessandretti R (2015). Zirconia as a dental biomaterial. Materials (Basel).

[4] Han A, Tsoi KHJ, Matinlinna PJ, Chen Z (2017). Influence of grit-blasting and hydrofluoric acid etching treatment on surface characteristics and biofilm formation on zirconia. Coatings.

[5] Guazzato M, Quach L, Albakry M, Swain MV (2005). Influence of surface and heat treatments on the flexural strength of Y-TZP dental ceramic. J Dent.

[6] Paravina RD, Roeder L, Lu H, Vogel K, Powers JM (2004). Effect of finishing and polishing procedures on surface roughness, gloss and color of resin-based composites. Am J Dent.

[7] Manojlovic D, Lenhardt L, Milićević B, Antonov M, Miletic V, Dramićanin MD (2015). Evaluation of staining-dependent color changes in resin composites using principal component analysis. Sci Rep.

[8] Erdemir U, Yildiz E, Saygi G, Altay NI, Eren MM, Yucel T (2016). Effects of energy and sports drinks on tooth structures and restorative materials. World J Stomatol.

[9] Tahmassebi JF, Duggal MS, Malik-Kotru G, Curzon ME (2006). Soft drinks and dental health: a review of the current literature. J Dent.

[10] West NX, Hughes JA, Addy M (2001). The effect of pH on the erosion of dentine and enamel by dietary acids in vitro. J Oral Rehabil.

[11] Berns RS (2019). Billmeyer and Saltzman's Principles of Color Technology. John Wiley & Sons, New york.

[12] Da Silva DL, Mattos CT, De Araújo MVA, De Oliveira RAC (2012). Color stability and fluorescence of different orthodontic esthetic archwires. Angle Orthod.

[13] Klimek J, Hellwig E, Ahrens G (1982). Fluoride taken up by plaque, by the underlying enamel and by clean enamel from three fluoride compounds in vitro. Caries Res.

[14] Nimeroff I, Yurow JA (1965). Degree of metamerism. J Opt Soc Am.

[15] Colombo M, Cavallo M, Miegge M, Dagna A, Beltrami R, Chiesa M, Poggio C (2017). Color stability of CAD/CAM zirconia ceramics following exposure to acidic and staining drinks. J Clin Exp Dent.

[16] Haralur SB, Raqe SAN, Alhassan Mujayri F (2019). Effect of hydrothermal aging and beverages on color stability of lithium disilicate and zirconia based ceramics. Medicina.

[17] Derafshi R, Khorshidi H, Kalantari M, Ghaffarlou I (2017). Effect of mouthrinses on color stability of monolithic zirconia and feldspathic ceramic: an in vitro study. BMC Oral Health.

[18] Spyropoulou PE, Kamposiora P, Eliades G, Papavasiliou G, Razzoog ME, Bayne SC (2016). Cyclic loading effect on color stability of unshaded versus shaded zirconia. J Esthet Restor Dent.

[19] Wegner SM, Kern M (2000). Long-term resin bond strength to zirconia ceramic. J Adhes Dent.

[20] Ourahmoune R, Salvia M, Mathia TG, Mesrati N (2014). Surface morphology and wettability of sandblasted PEEK and its composites. Scanning.

[21] Gawriolek M, Sikorska E, Ferreira LF (2012). Color and luminescence stability of selected dental materials in vitro. J Prosthodont.

[22] Badra VV, Faraoni JJ, Ramos RP, Palma-Dibb RG (2005). Influence of different beverages on the microhardness and surface roughness of resin composites. Oper Dent.

[23] Da Silva DL, Mattos CT, Simao RA, de Oliveira Ruellas AC (2013). Coating stability and surface characteristics of esthetic orthodontic coated archwires. Angle Orthod.

[24] Pratomo AH, Triaminingsih S, Indrani DJ (2018). Effect on tooth discoloration from the coffee drink at various smoke disposal during coffee bean roasting. J Phys Conf Ser.

[25] Schulze KA, Marshall SJ, Gansky SA, Marshall GW (2003). Color stability and hardness in dental composites after accelerated aging. Dent Mater.

